# Supplementary material for: Increased Risk of Fractures and Use of Proton Pump Inhibitors in Menopausal Women: A Systematic Review and Meta-Analysis
Source: Int J Environ Res Public Health. 2022 Oct 19;19(20):13501. doi: 10.3390/ijerph192013501 (PMC9603342; doi:10.3390/ijerph192013501)
Supplement: Supplementary file 1 [file ijerph-19-13501-s001.zip › ijerph-1900052-supplementary.pdf]

Supplementary Material

Table S1. Assessment of the methodological quality of selected studies based on the Newcastle-Ottawa scale

References	Kind of study	Selection	Comparability	Results	Total
Roux et al [16]	Prospective cohort	4	2	3	9
Gray et al [17]	Prospective cohort	4	2	3	9
Khalili et al [12]	Prospective cohort	3	2	3	8
Lewis et al [18]	Prospective cohort	4	1	3	8
Moberg et al [19]	Prospective cohort	4	2	3	9

The methodological quality of the study was assessed according to the Newcastle Ottawa Scale for cohort studies. The methodological quality score of the cohort studies was calculated in three components: group selection (0 - 4 points), comparability (0 - 2 points), and outcome (0 - 3 points).

Table S2. Results of selected studies relating the occurrence of fractures and the use of PPIs with other interfering variables

References	Outcomes	Vitamin use	Other drug classes	Overweight or obesity	Low weight	Smoking	Hormone therapy
**Roux et al** [16]	Patients using omeprazole history of mild fractures and higher prevalence of vertebrae fractures than non-users	No	Yes	No	No	No	Yes
**Gray et al** [17]	PPI not related to hip fracture risk, but increased risk of spine, forearm or wrist fractures and total fractures	No	Yes	Yes	No	No	No
**Khalili et al** [12]	Increased risk of hip fracture compared to users who did not use PPI regularly	No	Yes	Yes	No	No	Yes
**Lewis et al** [18]	Long-term therapies with PPIs are linked to increased risk of fractures, falls and hospitalizations	Yes	Yes	No	Yes	No	No
**Moberg et al** [19]	Menopausal women that are PPI user have an increase risk of fractures.	No	Yes	Yes	Yes	Yes	Yes
